# Comparative Evaluation of Chemical Composition, Phenolic Compounds, and Antioxidant and Antimicrobial Activities of Tropical Black Bolete Mushroom Using Different Preservation Methods

**DOI:** 10.3390/foods10040781

**Published:** 2021-04-05

**Authors:** Jaturong Kumla, Nakarin Suwannarach, Keerati Tanruean, Saisamorn Lumyong

**Affiliations:** 1 Department of Biology, Faculty of Science, Chiang Mai University, Chiang Mai 50200, Thailand; suwan.462@gmail.com (N.S.); scboi009@gmail.com (S.L.); 2 Research Center of Microbial Diversity and Sustainable Utilization, Chiang Mai University, Chiang Mai 50200, Thailand; 3 Biology Program, Faculty of Science and Technology, Pibulsongkram Rajabhat University, Phitsanulok 65000, Thailand; keerati.t@psru.ac.th; 4 Academy of Science, The Royal Society of Thailand, Bangkok 10300, Thailand

**Keywords:** antioxidant properties, edible mushroom, nutrition, phenolic compounds, preservation

## Abstract

Tropical black bolete, *Phlebopus portentosus*, provides various nutritional benefits and natural antioxidants to humans. In this study, the chemical composition, phenolic compounds, and antioxidant and antimicrobial activities of fresh mushroom samples and samples stored for a period of one year using different preservation methods (drying, brining, and frozen) were investigated. The results indicated that the brining method significantly reduced the protein and fat contents of the mushrooms. The polyphenol and flavonoid contents of the frozen sample were not significantly different from that of the fresh sample. The results revealed that an inhibition value of 50% (IC_50_) for the 2,2-diphenyl-1-picrylhydrazyl (DPPH) assay of the extract of the dried and frozen samples was not statistically different from that of the fresh sample. The IC_50_ value of 2,2′-azino-bis(3-ethylbenzothiazoline-6-sulfonic acid (ABTS) assay and ferric reducing antioxidant power (FRAP) value in the extract of the frozen sample were not found to be significantly different from those of the fresh sample. Furthermore, the lowest degree of antioxidant activity was found in the extract of the brined sample. Additionally, the antimicrobial activities of the extracts of the fresh and frozen samples were not significantly different and both extracts could have inhibited the growth of all tested Gram-positive bacteria and *Pseudomonas aeruginosa*.

## 1. Introduction

Varieties of certain cultivated edible mushroom genera, such as *Agaricus*, *Auricularia*, *Lentinus*, *Lentinula, Pleurotus*, *Tramella,* and *Volvariella*, account for the majority of the mushrooms traditionally consume by humans [1,2,3]. However, wild mushrooms have recently become increasingly consumed in our diets due to the recognition of their nutritional and pharmacological characteristics [4,5,6]. Ectomycorrhizal (ECM) mushrooms are a group of wild mushrooms, of which about 2500 recorded species have been defined as edible [7,8]. ECM mushrooms are a good source of essential dietary nutrients, such as carbohydrates, fiber, protein, minerals, and vitamins, all of which make them a valuable food resource for humans [9,10,11]. Apart from the use of ECM mushrooms as a source of food, they can be important in medicine due to their antibiotic, anticancer, antidiabetic, antioxidant, and increased immune response effects [5,12,13,14,15]. Currently, the consumption of edible ECM mushrooms has been increasing and this is mainly attributed to their elevated market value and an increase in consumer demand. Some of them, such as chanterelles (*Cantharellus* spp.), king boletes (*Botetus edulis*), matsutakes (*Tricholoma matsutake*), morels (*Morchella* spp.), and truffles (*Tuber* spp.), are among the world’s most expensive foods [7,8,16,17]. Generally, ECM mushrooms are a seasonal and highly perishable crop. After harvesting, various physiological and morphological characteristics of ECM mushrooms change, which makes them unacceptable for human consumption and limits their ability to be effectively marketed. The shelf-life of several ECM mushrooms is only about 2–5 days depending upon the mushroom species [18,19]. Fresh mushrooms are best stored unwashed in brown paper bags in the refrigerator, preferably on the lowest shelf. However, many methods have been employed to improve the preservation of these mushrooms and enhance their shelf-life. The most common methods include brining, canning, drying, freezing, and radiation [20,21]. For the purposes of marketing, popular edible ECM mushrooms, known as chanterelles (*Cantharellus* spp.), king boletes (*B. edulis*), morels (*Morchella* spp.), and truffles (*Tuber* spp.), are commonly preserved through the different processes [22,23,24].

Thailand is home to a great diversity of wild edible mushroom species that are most abundant during the wet season (mid-May to October) of each year [9,25]. Normally, local farmers collect edible wild ECM mushrooms for both consumption and sale in local, roadside, or city markets. Preliminary investigations at small local markets have revealed the availability of many genera of wild, edible ECM mushrooms such as *Amanita*, *Astraeus*, *Boletus, Cantharellus*, *Lactarius*, *Phlebopus,* and *Russula* [9,16,26,27]. The tropical black bolete (TBB), *Phlebopus portentous*, is a wild edible ECM mushroom that is known to be distributed throughout Asia, Australasia, and Mexico [9,16,28,29,30,31]. This mushroom is widely consumed in southern China, Laos, Myanmar, and northern and northeastern Thailand. In northern Thailand, this mushroom begins producing fruiting bodies at the end of the hot season and does so until the early wet season (May–July). On occasion, a second flush occurs at the end of the wet season (October). The market price for this mushroom is high and it is usually offered at 150–300 Baht/kg (5–10 USD/kg) [9,16]. This bolete produces large fruiting bodies and its texture is similar or even better than that of the king bolete, which is an important edible ECM mushroom in the European market [16,32]. This mushroom variety is known to contain high amounts of protein and is also known to possess a range of medicinal properties [9,13]. The study aimed to investigate the chemical composition, phenolic compounds, and the antioxidant and antimicrobial activities of TBB samples that had been stored for a one-year period using different preservation methods (drying, brining, and freezing). Generally, the preserved mushrooms will have been stored for approximately 2–3 years depending upon the preservation method [18,19,20,21]. However, it has been recommended that they should be consumed no later than one year from the sell date on the package. Thus, we selected a storage period of one year for this study. The results of this study will provide valuable information on the nutrient contents, phenolic compounds, and antioxidant and antimicrobial potential of this mushroom through the use of different preservation methods. The results could also be used to further enhance relevant strategies for potential commercial development.

## 2. Materials and Methods

### 2.1. Source of Mushroom and Sample Preparation

**Figure 1:**
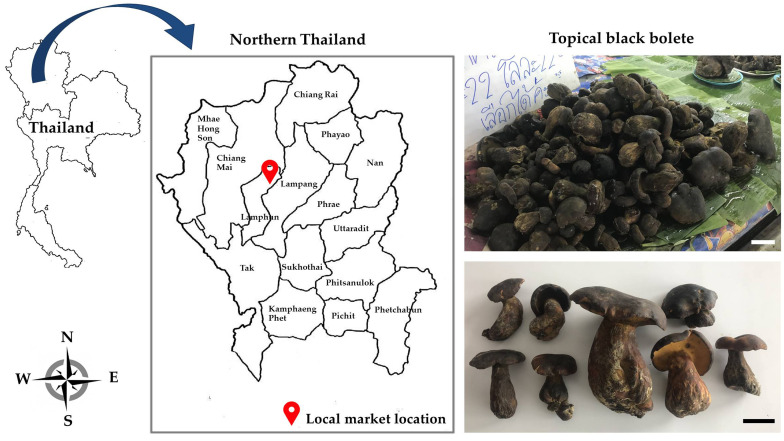
Local market location and fresh fruiting bodies of the tropical black bolete. Scale bar = 5 cm.

Fresh fruiting bodies of TBB were bought from local markets at Mae Ta District, Lamphun Province, northern Thailand in 2018 (Figure 1). The fruiting bodies of TBB were characterized by caps: convex to subconvex, centrally depressed, 3.0–15 cm in diameter, olive brown to dark brown; stipes: 3.5–7.0 × 7.0–10 cm, clavate or tapering to apex, concolored with cap; tubes under cap: yellow to yellowish-orange; contexts: soft, yellow (Figure 1). The fresh fruiting bodies were transferred to a laboratory within 24 h of being harvested, and the soil was removed. Mushroom sample was prepared following the method described by Tolera and Abera [33] with some modifications. Whole fruiting bodies were washed under running tap water and drained. The cleaned fruiting bodies were directly sliced into pieces of 4–5 mm thickness using a stainless steel knife. Sliced samples were then used in each preservative treatment.

### 2.2. Preservation Method

#### 2.2.1. Drying

Briefly, the sliced fresh mushroom samples were loaded onto trays and placed in a commercial food dryer at a temperature of 45 °C for 72 h (completely dried). The moisture content was then recorded. The samples were placed in zip-lock plastic bags and kept at room temperature (28 ± 2 °C) in the darkness.

#### 2.2.2. Brining

Sliced fresh mushroom samples were boiled in hot water (95 to 99 °C) for 5 min for the purposes of blanching, according to the method described by Lungu et al. [34]. They were then drained and placed immediately in cold water to cool. Subsequently, 100 g of the boiled product was placed in each 300 mL glass bottle. The brine solution (2.5% salt concentration) was then boiled. After cooling, the brine solution was added to glass bottles that contained the sliced mushrooms until the mushrooms were completely covered. A layer of rice bran oil was added, and they were then steamed for one hour. After cooling, the glass bottles were kept at room temperature in the darkness.

#### 2.2.3. Freezing 

The sliced fresh mushroom samples (20 pieces) were placed in each commercial zip-lock plastic bag (25 × 35 cm) and frozen at −20 °C in a freezer.

### 2.3. Proximate Composition Analysis

The proximate composition of each mushroom sample was identified as carbohydrate, protein, fat, ash, and fiber according to the Association of Official Analytical Chemists (AOAC) method [35] at Central Laboratory (Chiang Mai, Thailand) Company Limited (Chiang Mai, Thailand). The analysis was performed on the samples of each treatment in five replications. The obtained data were compared with the results of the fresh sample.

### 2.4. Preparation of Mushroom Extracts

Fresh samples were oven-dried in an oven at 45 °C for 72 h before being used. Additionally, all one-year storage samples, except the dried sample, were also oven-dried at 45 °C for 72 h. Each dried sample was ground using a Waring blender (New Hartford, CT, USA). Ground mushroom samples (20 g) were extracted with 200 mL of methanol at 25 °C and at 150 rpm for 24 h following the method described by Kaewnarin et al. [13]. The extracts were then sonicated using a Crest Ultrasonicator (Ewing Township, NJ, USA) and filtered through Whatman’s No. 1 filter paper. The residue was then re-extracted twice with methanol as has been described above. The methanol extract was then rotary evaporated at 40 °C to dryness. The extracts were stored at −20 °C for further use.

### 2.5. Determination of Phenolic Compounds

#### 2.5.1. Total Polyphenol Content

The total polyphenol content was estimated using the protocol previously described by Thitilertdecha et al. [36] with some modifications. The mushroom extract of each sample at 0.25 mL was mixed with 2.5 mL deionized water and 0.5 mL of Folin−Ciocalteu reagent. After 5 min, 0.5 mL of Na_2_CO_3_ (20% *w*/*v*) was added. The reaction mixture was then incubated at room temperature for one hour. Analysis of total polyphenol content was carried out by measurement of the absorbance at 760 nm. The standard curve of gallic acid was used to calculate the total polyphenol content of the samples (25–100 μg/mL; y = 0.006x; R^2^ = 0.997). The results were expressed as milligrams of gallic acid equivalent per gram of dry weight (mg GAE/g dw). Five replications were performed for the samples of each treatment.

#### 2.5.2. Total Flavonoid Content

Total flavonoid content was determined according the protocol established by Kaewnarin et al. [37], and quercetin was used as the standard flavonoid. Next, 0.5 mL of the extract was mixed with 2 mL of distilled water, and then 0.15 mL of NaNO_2_ (50 g/L) was added. After 5 min, 0.15 mL of AlCl_3_ (100 g/L) was added. The reaction was mixed and incubated at room temperature for 15 min. Absorbance was measured at 415 nm. The standard curve of quercetin was then used to calculate the total flavonoid content in the samples (25–300 μg/mL; y = 0.004x; R^2^ = 0.996). The total flavonoid contents were expressed as milligrams quercetin equivalent per gram of dry weight (mg QE/g dw). The data were presented as the average of five replications of each treatment.

### 2.6. Antioxidant Assay

#### 2.6.1. DPPH Scavenging Assay

The DPPH (2,2-diphenyl-1-picrylhydrazyl) scavenging ability was determined according to the method described by Gülçin et al. [38]. Initially, 0.5 mL of different concentrations of each extract sample was mixed with 1.5 mL of the 0.1 mM DPPH solution in methanol. A control mixture was prepared that consisted of 0.5 mL of methanol and 1.5 mL of the DPPH solution. The mixtures were incubated at room temperature for 30 min in the dark. Subsequently, the absorbance was measured at 517 nm. The percentage discoloration of DPPH radical of the samples was then calculated according to the following formula: percentage inhibition = (A_o_ − A_s_/A_o_) × 100, where A_o_ represents the absorbance of the control and A_s_ represents the absorbance of the mixture containing the extract sample. An inhibition concentration of 50% (IC_50_) was expressed as a calculation from the plot of percentage inhibition against the extract concentration. Samples of each treatment were analyzed in five replications.

#### 2.6.2. ABTS Scavenging Assay

ABTS (2,2′-azino-bis(3-ethylbenzothiazoline-6-sulfonic acid) scavenging activity was determined according to the method described by Re et al. [39] with some modifications. The solution of ABTS cation chromophore was prepared by facilitating a reaction between 100 mL of the 7.0 mM ABTS solution and 100 mL of 2.45 mM K_2_S_2_O_8_. The solution was kept in darkness at 25 °C for 16 h. The ABTS solution was adjusted to an absorbance value of 0.70 ± 0.2 at 734 nm by dilution with the phosphate buffer (50 mM, pH 7.4) before being used. Next, 2.9 mL of the ABTS solution was mixed with 0.1 mL of different concentrations of each extract sample. A mixture composed of the ABTS solution and methanol was used as the control. The mixtures were incubated at room temperature for 30 min in the dark. The absorbance value of the mixture was measured at 734 nm. The percentage of discoloration of the ABTS radical was calculated according to the formula previously described in the DPPH assay, as has been described above. The IC_50_ value was then calculated, and five replications were completed for the samples of each treatment.

#### 2.6.3. FRAP Assay

FRAP (ferric reducing antioxidant power) assay was performed according to the protocol described by Li et al. [40]. The FRAP reagent was prepared using a mixture containing 10 mM 2,4,6-tripyridyl-s-triazine solution in 20 mL of 40 mM HCl, 20 mL of 20 mM ferric (III) chloride, and 5 mL of 300 mM acetate buffer (pH 3.6). Each 0.1 mL of extract was mixed with 1.5 mL of FRAP reagent and 1.4 mL of acetate buffer (300 mM, pH 3.6). The mixture was then incubated at room temperatures for 30 min in the dark. The absorbance at 593 nm was measured. Gallic acid was used to calculate the standard curve (10–100 μg/mL; y = 0.014x; R^2^ = 0.995), and the FRAP value was calculated as mg GAE/g dw. The data were presented as the average of five replications of each treatment.

### 2.7. Antimicrobial Assay

#### 2.7.1. Microorganisms

The microorganisms used in this study consisted of 8 strains of Gram-positive bacteria (*Bacillus cereus*, *B*. *subtilis*, *Enterococcus faecalis* ATCC29212*, Listeria monocytogenes, Micrococcus luteus*, Methicillin-resistant *Staphylococcus aureus*, *Sta*. *aureus* ATCC29213, and *Streptococcus pneumoniae* ATCC49699), 10 strains of Gram-negative bacteria (*Escherichia coli* ATCC35218, *E*. *coli* ATCC25922, *E*. *coli* O157:H7, *Klebsiella pneumonia, Proteus mirabilis*, *Pr*. *vulgaris*, *Pseudomonas aeruginosa* ATCC27859, *Ps*. *fluorescens*, *Salmonella typhi,* and *Salmonella* sp. group D), and 2 yeast strains (*Candida albicans* and *Cryptococcus neoformans*). All tested microorganisms were obtained from the Sustainable Development of Biological Resources Laboratory, Department of Biology, Faculty of Science and the Central and Diagnostic Laboratory, Maharaj Nakorn Chiang Mai Hospital, Faculty of Medicine Chiang Mai University, Thailand. Bacteria and yeast samples were grown and maintained on nutrient agar (NA) and yeast extract peptone dextrose agar (YPDA) slants, respectively. The inoculated slants were incubated at 37 °C.

#### 2.7.2. Determination of Antimicrobial Activity

The antimicrobial activity of the mushroom extracts was determined by paper disc diffusion assay [41,42]. Bacteria and yeast samples were cultured in a nutrient broth at 37 °C and yeast extract peptone dextrose broth at 30 °C, respectively. Their cultivation was then achieved on an orbital shaker at 125 rpm for 24 h. The cell density of both test microorganisms was then adjusted to 10^8^ CFU/mL that corresponded to the 0.5 McFarland standard. All prepared suspensions of the test microorganisms were swabbed on their respective agar media including NA (for the bacteria test) and YPDA (for the yeast test). Sterile paper discs (8 mm in diameter) were impregnated with 40 μL of each mushroom extract at a concentration of 100 mg/mL. The discs were allowed to dry and then placed on the agar surface of the tested plate. Discs with the methanol were used as the negative control, while standard antibiotics such as ampicillin, streptomycin, and nystatin at concentrations of 10 mg/mL were used as positive controls. The plates were incubated at 37 °C for 24 h. After incubation, the zone of inhibition appeared around the discs and was measured and recorded. Five replications were made for each sample extract obtained from each treatment.

### 2.8. Statistical Analysis

The data collected were subjected to one-way analysis of variance (ANOVA) by SPSS program version 16.0 for Windows. Tukey’s test was used to determine significant differences (*p* < 0.05) between the mean values.

## 3. Results and Discussion

### 3.1. Characteristics of Mushroom Samples

**Figure 2:**
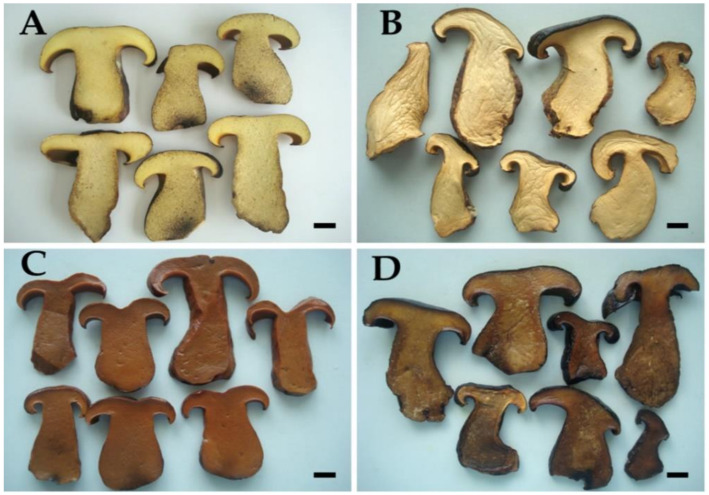
Fresh and one-year storage of tropical black bolete mushroom samples. (**A**) Fresh samples. (**B**) Dried samples. (**C**) Brined samples. (**D**) Frozen samples. Scale bar = 1 cm.

Fresh TBB samples contained a moisture content of 82.7 ± 2.0% on a wet-weight basis. Our results agreed with the findings of previous studies that found that the moisture content in fresh bolete mushrooms ranged from 70% to 93% depending upon the bolete species [43,44,45,46,47]. To extend the availability and shelf-life of TBB, a preservation method is recommended. Methods of drying, brining, and freezing were used in this study. Fresh samples and preserved samples after one year of storage were observed, and the pictures depicting the different treatments are shown in Figure 2. Based on a visual observation, the colors of the dried, frozen, and brined samples were cream, light brown, and brown, respectively. It was found that the color of the frozen sample was similar to that of the fresh sample. Additionally, the frozen sample also had a pronounced flavor that was similar to that of the fresh sample. However, both the dried and brined samples revealed a slight change in flavor. These results are in accordance with those of prior studies that reported that different preservation methods can lead to flavor and color changes in mushrooms [20,43,48].

### 3.2. Proximate Composition

The amounts of ash, carbohydrate, protein, fat, and fiber of the different mushroom samples are shown in Table 1, all of which were within the ranges described in previous reports with regard to the ash (6.7%–27.6%), carbohydrate (33.3%–65.1%), protein (14.0%–36.3%), fat (0.4%–9.5%), and fiber (3.1%–14.7%) contents found in several edible ECM mushrooms including boletes [9,10,11,42,49,50,51]. It was found that the drying, brining, and freezing methods did not affect the ash, carbohydrate, and fiber contents of the samples. Notably, the drying and freezing methods did not cause a significant change in protein content when compared with the fresh sample. However, the lowest protein and fat contents were found in the brined sample. This result is in full agreement with the results of previous studies that indicated that the brining method decreased the protein and fat contents of the mushroom [20,34,48]. Pagoń et al. [52] found that the brining method significantly decreased the protein content in boletes (*B. edulis* and *Suillus luteus*) after storage at room temperature for four weeks. Previous studies have reported that lower protein and fat contents in the brined samples can be explained by the much greater amounts of fat, protein, and amino acid solubilizations that exist as a result of employing the brining method [34,48,53,54].

### 3.3. Determination of Phenolic Compounds

Total polyphenol and flavonoid contents of all methanol extracts of TBB samples are shown in Table 2. The total polyphenol and flavonoid contents in the fresh samples were recorded at 30.10 ± 1.04 mg GAE/g dw and 1.69 ± 0.71 mg QE/g dw, respectively. The polyphenol content obtained in this study was within the range of the polyphenol (1.44–38.44 mg GAE/g dw) and flavonoid (0.03–2.54 mg QE/g dw) contents of other edible ECM mushrooms recorded in previous reports [12,13,14,55,56], depending upon the mushroom species. It was found that the total polyphenol and flavonoid contents in the preserved samples varied according to different preservation methods. A significant decrease in the polyphenol and flavonoid contents was observed in both the dried and brined samples. The lowest total polyphenol (18.44 ± 0.54 mg GAE/g dw) and flavonoid (0.37 ± 0.31 mg QE/g dw) values were observed in the brined samples. These results were supported by the findings of previous studies that reported that the decrease in the phenolic compound contents in the dried and brined samples could be explained by the application of thermal treatment via boiling, canning, drying, and blanching. These thermal treatments increased the extractability of both the phenolic compounds and the phenolic compounds that were possibly degraded during storage as a result of sensitivity oxidation and solubilization [34,57,58]. Ganguli et al. [59] and Pagoń et al. [52] reported that the loss of total polyphenol contents in white bottom mushrooms (*Agaricus bisporus*) and boletes (*B. edulis* and *Sui. luteus*) were due to the blanching and brining processes, respectively. Nevertheless, the total polyphenol and flavonoid contents in the frozen samples examined in this study were not found to be significantly different when compared with the fresh sample. This result was in full agreement with that the findings presented in previous studies that reported that the freezing process can cause minimal amounts of destruction of the polyphenol and flavonoid compounds in fruits, some vegetables, and mushrooms [57,59]. In addition, the oven-dry process (45 °C for 72 h) used for preparation of the dried samples obtained from the brined and frozen samples may have affected their phenolic compound composition, as well as the total polyphenol and flavonoid compounds. Although the measurement of total polyphenol and flavonoid compounds was carried out using several methods in this study, other techniques such as high-performance liquid chromatography and mass spectrometry should be applied to account for changes to the phenolic profile in each sample that occurred during storage.

### 3.4. Antioxidant Assay

Several edible ECM mushroom genera, namely, *Amanita*, *Astraeus*, *Boletus*, *Cantharellus*, *Lactarius*, *Leccinum*, *Phlebopus*, *Russula*, *Tricholoma*, *Suillus,* and *Xerocomus*, have been reported to contain higher amounts of secondary metabolites, which are indicative of multiple biological effects, including the antioxidant activity that can act according to different mechanisms of action for each mushroom species [12,13,14,47,56,60]. Thus, a single method cannot fully estimate the antioxidant capacity of these mushrooms. Therefore, ABTS, DPPH, and FRAP assays were used to evaluate the possible antioxidant activity of the methanolic extracts of different samples of TBB in this study. ABTS and DPPH values were determined by assessing the scavenging activity on ABTS and DPPH radicals (by measuring the decrease in ABTS and DPPH radical absorption after exposure to radical scavengers), respectively. Furthermore, FRAP assay was used to measure the conversion of the ferric form (Fe^3+^) to the ferrous form (Fe^2+^). Results are shown in Table 3. 

In the DPPH and ABTS radical scavenging systems, the extract concentrations providing IC50 values were of particular interest. The IC50 value is a comparable parameter widely used to measure the potency of antioxidant activity of test samples. Thus, the lower IC50 value indicated the higher antioxidant activity. In the DPPH scavenging system, the results indicated that the IC50 values of the DPPH assay varied for each sample. The lowest IC50 value of the DPPH activity was observed in the extract of the fresh sample (2.11 ± 0.08 mg/mL), which was not found to be statistically different from that of the dried (2.20 ± 0.60 mg/mL) and frozen samples (2.14 ± 0.72 mg/mL). However, they were significantly lower than that of the brined sample (3.65 ± 0.48 mg/mL). In the ABTS scavenging system, all extracts showed positive results in terms of the ABTS assay and the IC50 values, which varied from 1.30 to 2.85 mg/mL. The lowest IC50 value of ABTS activity was observed in the extract of the fresh sample, followed by the frozen sample and the dried sample.

In the ABTS scavenging system, all extracts showed positive results in terms of the ABTS assay and the IC_50_ values, which varied from 1.30 to 2.85 mg/mL. The lowest IC_50_ value of ABTS activity was observed in the extract of the fresh sample, followed by the frozen sample and the dried sample. However, the highest IC_50_ value of ABTS activity was found in the extract of the brined sample. In the FRAP system, the FRAP values of the extract of the fresh sample (7.86 ± 0.11 mg GAE/g dw) and frozen sample (7.52 ± 0.38 mg GAE/g dw) were significantly higher than those of the extracts obtained from the other samples. Moreover, the lowest FRAP value was observed from the extract of the brined sample (3.81 ± 0.46 mg GAE/g dw).

Our results are similar to those of previous studies that reported that the methanolic extract of ECM mushrooms, including boletes, revealed notable DPPH, ABTS, and FRAP activities [13,14,47,60,61]. The results indicate that different preservation methods affected the antioxidative activities of the samples. These findings are supported by those of previous studies that reported that the preservation method can influence the antioxidant activity of the mushroom [57,60]. In this study, the brining method led to the highest degree of decreasing value of antioxidant activity. This outcome is in concordance with Ganguli et al. [59] and Muruke [62], who found that the fresh white bottom mushroom and the oyster mushroom (*Pleurotus cystidiosus*) revealed the strongest degrees of antioxidant activity when compared to the brined mushrooms. Moreover, Pagoń et al. [52] reported that the DPPH, ABTS, and FRAP activities in the brined samples of *B. edulis* and *Sui. luteus* after four weeks of storage were lower than in the fresh samples. In this study, the freezing process maintained the antioxidant activity in the TBB sample during storage. This result was supported by the findings of previous studies that indicated that the act of freezing did not induce or even slightly change the degree of antioxidant activity in certain mushrooms (e.g., desert truffles, chanterelles, sweet tooth mushrooms, shitake mushrooms, and chestnut mushrooms) [57] and vegetables (broccoli florets, cauliflower, green asparagus, and kale leaves) [63,64,65,66].

### 3.5. Antimicrobial Assay

Antimicrobial activity of the crude extracts of the fresh and preserved samples after one year of storage was investigated in terms of the inhibition zone against eighteen strains of pathogenic bacteria and yeast. The diameter of the inhibition zone is shown in Table 4. It was found that ampicillin and streptomycin effectively inhibited all tested bacteria. The diameter of the inhibition zone of ampicillin and streptomycin was significantly larger than that of the mushroom extracts. The result revealed that the inhibition zone varied according to the differences in the extract samples. The extracts of the fresh and frozen samples could effectively inhibit the growth of all tested Gram-positive bacteria. However, the extracts of the brined and dried samples could not inhibit the growth of *B*. *subtilis* and *L*. *monocytogenes*. The growth of *B*. *cereus* and *M*. *luteus* could not be inhibited by the extract of the brined sample. The extract of the fresh sample displayed the largest diameter of the inhibition zone over the other extracts. Most of the extracts showed no antimicrobial activity against all Gram-negative bacteria, except for the extract of the fresh and frozen samples that could inhibit the growth of *Ps*. *fluorescens*. However, all extracts displayed no antimicrobial activity against all the yeast samples at the concentration values used. Similarly, antimicrobial potential has been observed in the extracts of the other edible ECM mushrooms in that they displayed greater activity against Gram-positive bacteria than against Gram-negative bacteria and yeast specimens [12,14,42,55]. Kosanić et al. [56] and Smolskaitė et al. [61] reported that the intensity of the antimicrobial activity was dependent upon the mushroom species, its concentration value, and the tested microorganisms. Our results indicate that different preservation methods can influence the antimicrobial activity of the mushroom samples.

Additionally, it was found that freezing serves as a suitable preservation method in maintaining the antimicrobial activity in the TBB sample during storage. These results were supported by the findings of previous studies that found that the perseveration process does play a significant role in reducing the antimicrobial activity of mushrooms. Additionally, it was found that the high temperatures reached during the preservation process, for exampl, drying, boiling, and blanching, could partially degrade the antimicrobial active compounds in mushrooms [67,68,69]. Furthermore, a study conducted by Janeš et al. [70] found that the extracts of several dried mushroom samples displayed lower antibacterial activity than extracts of the frozen samples. Based on our results, the average amount of total polyphenol content of the extracts obtained from fresh, dried, brined, and frozen samples for antibacterial assay was 3.00, 2.66, 1.84, and 2.89 mg GAE at a concentration of 100 mg/mL, respectively. At a concentration extract of 100 mg/mL, the average value of total flavonoid content contained in the extracts obtained from the fresh, dried, brined, and frozen samples was 0.26, 0.20, 0.06, and 0.25 mg QE, respectively. It was found that the high antibacterial potential possessed in the extracts of the fresh and frozen samples could be related to their high total polyphenol and flavonoid contents. 

## 4. Conclusions

TBB is considered a nutritional mushroom with a number of health benefits. Therefore, the ability to extend the shelf-life of this mushroom is crucial to gaining an understanding of how different preservation methods could affect the mushroom’s nutrient composition and antioxidant and antimicrobial properties. In this study, three preservation methods were used, namely, brining, drying, and freezing, in order to determine the effect on nutrient composition, phenolic compound content, and antioxidant and antimicrobial properties in TBB. After one year of storage, the brined sample showed significant reductions in crude protein, fat, polyphenol and flavonoid contents, as well as antioxidant and antimicrobial properties, when compared with the dried, frozen, and fresh samples. The outcomes of this study demonstrate that freezing could serve as a suitable preservation process by resulting in little or no change to the nutrient composition, phenolic compound content, and antioxidant and antimicrobial properties in samples after one year of storage. However, differences in phenolic profiles in terms of the amount of individual phenolic compounds present in the samples during storage should be determined in future studies. Moreover, mineral analysis, sensory evaluation, microbial contamination, and long-term storage of the preserved mushroom samples would be necessary, and to identify a suitable preservation method for potential commercial development.

## Figures and Tables

**Table 1:** Proximate composition on a dry basis (% dry weight) of different tropical black bolete samples.

Sample Type	% Dry Weight					
	Moisture	Ash	Crude Protein	Fat	Fibre	Carbohydrate
Fresh sample	7.2 ± 0.1 a	9.6 ± 0.2 a	19.6 ± 0.4 a	1.0 ± 0.1 a	6.3 ± 0.1 a	54.8 ± 0.7 a
Dried sample	7.1 ± 0.2 a	9.7 ± 0.2 a	19.3 ± 0.4 a	1.0 ± 0.2 a	6.2 ± 0.1 a	54.7 ± 0.6 a
Brined sample	7.0 ± 0.1 a	9.4 ± 0.1 a	17.1 ± 0.8 b	0.7 ± 0.1 b	6.2 ± 0.2 a	53.4 ± 0.5 a
Frozen sample	7.1 ± 0.2 a	9.6 ± 0.1 a	19.4 ± 0.3 a	0.9 ± 0.1 a	6.3 ± 0.2 a	54.6 ± 0.4 a

The results are mean ± standard deviation. Different letters in the same column are considered significantly different according to Tukey’s test (*p* < 0.05).

**Table 2:** Total polyphenol and flavonoid contents in the methanolic extracts obtained from different tropical black bolete samples.

Mushroom Extract	Total Polyphenol Content (mg GAE/g dw)	Total Flavonoid Content (mg QE/g dw)
Fresh sample	30.10 ± 1.04 a	1.69 ± 0.17 a
Dried sample	26.70 ± 0.63 b	1.32 ± 0.26 b
Brined sample	18.44 ± 0.54 c	0.37 ± 0.31 c
Frozen sample	29.06 ± 1.20 a	1.63 ± 0.24 a

The results are mean ± standard deviation. Different letters in the same column are considered significantly different according to Tukey’s test (*p* < 0.05).

**Table 3:** Antioxidant assay of the methanolic extracts obtained from different tropical black bolete samples.

Mushroom Extract	DPPH Assay (IC_50_, mg/mL)	ABTS Assay (IC_50_, mg/mL)	FRAP Assay (mg GAE/g dw)
Fresh sample	2.11 ± 0.80 b	1.30 ± 0.20 c	7.86 ± 0.24 a
Dried sample	2.20 ± 0.60 b	1.78 ± 0.27 b	5.53 ± 0.62 b
Brined sample	3.65 ± 0.48 a	2.85 ± 0.31 a	3.81 ± 0.46 c
Frozen sample	2.14 ± 0.72 b	1.72 ± 0.24 b	7.52 ± 0.38 a

The results are mean ± standard deviation. Different letters in the same column are considered significantly different according to Tukey’s test (*p* < 0.05).

**Table 4:** Diameter of inhibition zone of the methanolic extracts from different tropical black bolete samples and antimicrobial compounds.

Microorganism	Diameter of Inhibition Zone (mm)						
	Mushroom Extract (100 mg/mL)				Antimicrobial Compound (10 mg/mL)		
	Fresh Sample	Dried Sample	Brined Sample	Frozen Sample	Ampicillin	Streptomycin	Nystatin
**Gram-positive bacteria**							
*Bacillus cereus*	11.2 ± 0.8 c	10.5 ± 0.5 c	−	10.2 ± 0.5 c	28.3 ± 1.5 a	24.5 ± 1.2 b	NT
*Bacillus subtilis*	10.8 ± 0.7 c	−	−	10.2 ± 0.6 c	29.7 ± 1.2 b	34.1 ± 0.8 a	NT
*Enterococcus faecalis* ATCC29212	11.5 ± 1.5 c	11.1 ± 0.9 c	9.8 ± 0.2 c	11.1 ± 0.5 c	40.8 ± 1.0 a	22.2 ± 0.5 b	NT
*Listeria monocytogenes*	11.0 ± 0.5 c	−	−	10.5 ± 0.6 c	34.0 ± 1.7 a	22.7 ± 2.5 b	NT
*Micrococcus luteus*	11.5 ± 0.9 c	10.5 ± 0.8 cd	−	9.6 ± 0.5 d	17.5 ± 1.3 b	20.8 ± 1.0 a	NT
Methicillin-resistant *Staphylococcus aureus*	11.7 ± 0.6 c	11.0 ± 1.0 cd	9.9 ± 0.6 d	11.2 ± 0.7 c	16.8 ± 1.2 a	14.2 ± 0.8 b	NT
*Staphylococcus aureus* ATCC29213	11.5 ± 0.5 c	11.2 ± 0.8c	10.5 ± 0.8 c	11.0 ± 0.8 c	17.6 ± 0.5 b	26.5 ± 1.5 a	NT
*Streptococcus pneumoniae* ATCC49699	16.7 ± 1.2 c	13.2 ± 1.3 d	12.0 ± 1.0 d	14.2 ± 1.3 d	23.8 ± 1.3 b	30.8 ± 1.0 a	NT
**Gram-negative bacteria**							
*Escherichia coli* ATCC25922	−	−	−	−	18.0 ± 1.0 a	11.2 ± 1.0 b	NT
*Escherichia coli* ATCC35218	−	−	−	−	17.6 ± 0.5 a	11.8 ± 1.0 b	NT
*Escherichia coli* O157:H7	−	−	−	−	17.9 ± 0.2 a	12.5 ± 0.8 b	NT
*Klebsiella pneumoniae*	−	−	−	−	21.2 ± 1.0 a	11.5 ± 0.5 b	NT
*Proteus mirabilis*	−	−	−	−	34.2 ± 1.5 a	21.6 ± 1.0 b	NT
*Proteus vulgaris*	−	−	−	−	12.5 ± 0.7 b	27.3 ± 1.0 a	NT
*Pseudomonus fluorescens*	−	−	−	−	27.7 ± 2.0 a	10.2 ± 0.5 b	NT
*Pseudomonas aeruginosa* ATCC27859	9.83 ± 0.8 b	−	−	9.33 ± 0.6 b	20.2 ± 1.0 a	18.2 ± 1.0 a	NT
*Salmonella typhi*	−	−	−	−	30.7 ± 1.1 b	39.3 ± 1.1 a	NT
*Salmonella* sp. group D	−	−	−	−	20.7 ± 1.2 a	16.8 ± 0.8 b	NT
**Yeasts**							
*Candida albicans*	−	−	−	−	NT	NT	19.0 ± 1.0 a
*Cryptococcus neoformans*	−	−	−	−	NT	NT	20.7 ± 1.5 a

NT = not tested and (−) = no inhibition zone. The results are mean ± standard deviation. Different letters in the same row are considered significantly different according to Tukey’s test (*p* < 0.05).

## Data Availability

Data sharing not applicable.
